# An Enhanced Electroosmotic Micromixer with an Efficient Asymmetric Lateral Structure

**DOI:** 10.3390/mi7120218

**Published:** 2016-12-01

**Authors:** Teng Zhou, Hanlin Wang, Liuyong Shi, Zhenyu Liu, Sang Woo Joo

**Affiliations:** 1 Mechanical and Electrical Engineering College, Hainan University, Haikou 570228, Hainan, China; zhouteng@hainu.edu.cn (T.Z.); hanlinwang@gmail.com (H.W.); liuyongshi@gmail.com (L.S.); 2 School of Mechanical Engineering, Yeungnam University, Gyongsan 712-719, Korea; 3 Changchun Institute of Optics, Fine Mechanics and Physics (CIOMP), Chinese Academy of Science, Changchun 130033, Jilin, China

**Keywords:** microfluidics, micromixer, electroosmotic, active mixer, computational fluid dynamics

## Abstract

Homogeneous and rapid mixing in microfluidic devices is difficult to accomplish, owing to the low Reynolds number associated with most flows in microfluidic channels. Here, an efficient electroosmotic micromixer based on a carefully designed lateral structure is demonstrated. The electroosmotic flow in this mixer with an asymmetrical structure induces enhanced disturbance in the micro channel, helping the fluid streams’ folding and stretching, thereby enabling appreciable mixing. Quantitative analysis of the mixing efficiency with respect to the potential applied and the flow rate suggests that the electroosmotic microfluidic mixer developed in the present work can achieve efficient mixing with low applied potential.

## 1. Introduction

In many biochemical processes, efficient reagent mixing is often required [1,2,3,4]. In macro-scale devices, mixing of fluids usually relies mainly on convection effects, which tend to weaken as the geometrical scale decreases. In microfluidic channels, it is difficult to rapidly and homogenously mix different fluids due to the low Reynolds number associated with the microscale fluid flow. A number of micro mixing methods have been demonstrated to enhance the distance for interaction of the two samples and shorten the mixing distance required.

Many ingenious micromixers developed in recent years can be divided into two broad categories: passive [5,6,7,8,9] and active types [10,11,12,13,14,15,16,17,18]. Active micromixers utilize externally-induced mixing, such as electric [11,12,13,14,15,19], magnetic [18,20,21,22,23,24], acoustic [25], and moving parts [26] upon the flow field. Passive micromixers do not require external energy, except for that used to drive the flows [5,27,28]. Mostly, passive micromixers exploit the micromixers’ geometry to produce complex flow fields for effective mixing [27,28]. Compared to the passive method, the active type relies on external stimuli or energy to enhance the mixing of the reagent in the channel, and can show better mixing capabilities. Among the passive mixers, the electroosmotic flow (EOF) mixer is particularly effective in small channels, and suitable for fluids with low Reynolds numbers, and makes use of a DC voltage across the regions with positive and negative charges on the same substrate in a microchannel [10,11,12,15]. In general, the DC voltage can generate in-plane vortices, which can be simulated by two-dimensional (2D) computations.

In the present work, we present an EOF mixer with an efficient lateral structure with DC voltage across two coplanar groups of electrodes. The lateral structure is obtained based on the topography optimization method [8,29,30,31]. By adding four electrodes and adjusting their distribution, the channel with DC-EOF produces in-plane microvortices, helping the reagent mixing. Compared with traditional EOF mixers consisting of two circles [10], the efficiency of the proposed EOF micromixer with a lateral structure is significantly improved by the enhanced fluid motion. Besides, the electric field is not time-dependent, but stationary. The proposed mixer can thus be more compact and suitable for connection with other parts of a microfluidic system.

## 2. Mathematical Model and Numerical Method

**Figure 1:**
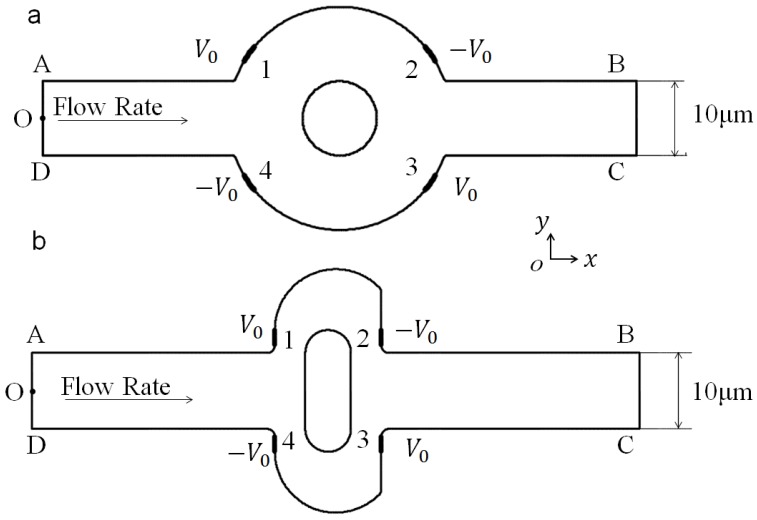
Configuration of (**a**) the original mixer structure with four symmetric electrodes on the wall of the mixing chamber; and (**b**) the optimized mixer. AD: inlet, BC: outlet, O: midpoint of boundary AD.

We consider a two-dimensional (2D) viscous flow in a microchannel with with a mixing region, as shown in Figure 1b. The channel depth-to-width ratio is assumed to be large enough for the three-dimensional effect of top and bottom to be neglected. The original mixer (shown in Figure 1a as a reference) is composed of two circular boundaries, while the optimized mixer of the present study is composed of two lateral structures, as designed by the authors using a topography optimization method.

In both mixers, an electric potential is applied externally from inlet AO and OD to grounded outlets BC. The incompressible electroosmotic flow in the fluid domain $\Omega_{f}$ is described by a coupled system of the Navier–Stokes equation, the Laplace equation for the electrical potential *ϕ* in in the channel, and the convection–diffusion equation for the concentration *c* of the dissolved substances in the fluid, as follows:

(1) $$\nabla \cdot [pI-\eta \left(\nabla u+\nabla u^{T}\right)]+\rho u\cdot \nabla u=f,$$

(2) ∇·u=0,

(3) $$\nabla^{2}\phi =0,$$

(4) u·∇c=∇·(D∇c),

where *ρ*, *η*, u, I, *p*, D are the fluid density, viscosity, velocity vector, identity tensor, pressure field, and diffusion coefficient, respectively. For water as the carrier fluid, ρ=1000 kg/m${}^{3}$ and η=0.001 Pa·s, with the diffusion coefficient D $=10^{-11}$ m${}^{2}$/s.

Along the inlets 1 and 2, a uniform flow is imposed,

(5) $$u=U_{01}n\;\mathrm{at}\;\Gamma_{AO},$$

(6) $$u=U_{02}n\;\mathrm{at}\;\Gamma_{OB}.$$

Here, $U_{01}$, $U_{02}$, and $n=(n_{x},n_{y})$ are the magnitude of the velocity of inlet AO, inlet OB, and the normal vector of the surface, respectively. In this work, $U_{01}$ = $U_{02}$ = $U_{0}$ is set. The thicknesses of the electric double layer (EDL) adjacent to the charged channel wall are very thin in comparison to the widths of the channel, and so the Smoluchowski slip boundary condition for Newtonian electroosmotic flow is applied on the charged channel wall AB and CD:

(7) $$u=u_{w}=\frac{\varepsilon_{f}\zeta_{w}}{\mu }\left(I-\mathrm{nn}\right)\cdot \nabla \phi \;\;\mathrm{at}\;\Gamma_{wall},$$

where $u_{w}$ is the fluid velocity on the channel wall, and $\varepsilon_{f}$ and $\zeta_{w}$ are, respectively, the fluid permittivity and the zeta potential of the channel wall, which are set to 80.2 and −0.1 V. The ambient pressure and no-traction condition are applied at outlet BC:

(8) $$\begin{array}{ccc} p & = & 0\;\mathrm{at}\;\Gamma_{outlet} \end{array}$$

(9) $$\begin{array}{ccc} \eta (\nabla u+\nabla u^{T})\cdot n & = & 0\;\mathrm{at}\;\Gamma_{outlet}. \end{array}$$

With the Laplace equation, the local electric field E can be calculated from the electric potential *ϕ* by

(10) $$E=-\nabla \phi \;\mathrm{in}\;\Omega_{f}.$$

In this chip, a potential shift is applied across the electrodes (1,3) and (2,4), so the boundary conditions for *ϕ* on the entrance and exits of the microchannel are

(11) $$\phi =\phi_{0}\;\mathrm{on}\;1,3$$

and

(12) $$\phi =-\phi_{0}\;\mathrm{on}\;2,4.$$

Other boundaries (including the channel wall $\left(\Gamma_{wall}\right)$, except on electrodes), inlet $\left(\Gamma_{inlet}\right)$ and outlet $\left(\Gamma_{outlet}\right)$ are electrically insulating, yielding

(13) n·∇ϕ=0onotherboundaries.

For the steady convection–diffusion equation, the concentrations at inlets 1 and 2 are specified as

(14) $$c=1\;\mathrm{mol}/m^{3}\;\mathrm{at}\;\Gamma_{inlet1}$$

(15) $$c=0\;\mathrm{mol}/m^{3}\;\mathrm{at}\;\Gamma_{inlet2},$$

while the condition of no species flux is imposed at channel walls,

(16) $$\left(cu-D\nabla c\right)\cdot n=0\;\mathrm{at}\;\Gamma_{wall}.$$

The boundary condition along the outlet is

(17) $$\left(D\nabla c\right)\cdot n=0\;\mathrm{at}\;\Gamma_{outlet}.$$

The mixing effect of the two flows with an anticipated distribution of the concentration near the outlet can be expressed using the least-square type optimization objective as [8,30,32], which is used as our mixing efficiency index,

(18) $$\sigma =\sqrt{\frac{\int_{\Gamma }{\left(c-\bar{c}\right)}^{2}}{L{\bar{c}}^{2}}},$$

where *c* is the concentration of the reagent, $\bar{c}$ denotes the average concentration, Γ is the cross line from which we measure the concentration; in this work, line BC is chosen as the cross line, and *L* is the length of the cross line. Complete mixing and complete segregation are then defined by σ=0 and σ=1, respectively. Therefore, the lower the value is, the better is the performance of the mixer.

The above system is solved numerically using the commercial finite element package COMSOL Multiphysics (Version 4.3a, COMSOL Group, Stockholm, Sweden). The coupled system of hydrodynamic, electrical, and concentration field is solved simultaneously.

## 3. Results and Discussion

In this section, the mixing process is presented using the concentration surface plot, the fluid streamlines, and electric potential lines for the original chip and revised chip with and without an electric field. Furthermore, the mixing efficiency index *σ* of chip versus the potential and the inlet mean velocity is analyzed to show the influence of the potential and the inlet mean velocity.

### 3.1. Mixing Process and Mechanism

**Figure 2:**
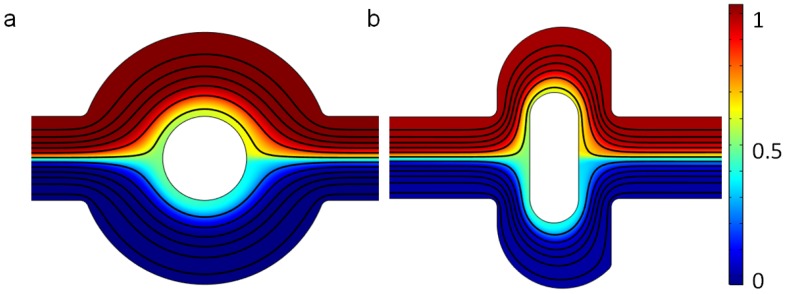
Fluid streamlines in the channel, and the concentration surface plot of (**a**) the original chip and (**b**) revised chip in the absence of an electric field and the mean inlet velocity $U_{0}=10^{-3}$ m/s. The unit for the concentration is mol/m${}^{3}$.

For two mixers, the two fluids in the channel are also well separated at the outlet, because the flow is laminar and the diffusion coefficient is very small when the electric field is not applied; that is, $V_{0}=0$ V, as shown in Figure 2. The mean inlet velocity of the two mixers is $U_{0}=10^{-3}$ m/s, and the concentrations at the upper and lower inlet are set as 1 mol/m${}^{3}$ and 0 mol/m${}^{3}$. The streamlines in Figure 2 demonstrate that there is no lateral flow, which would be vertical main flow with other fluctuations, owing to the slow mean velocity and low Reynolds number. Furthermore, the distance for the diffusion of two samples is not long enough. Thus the two solutions with different concentrations stay split, with an obvious interface in both the original and the revised chip.

**Figure 3:**
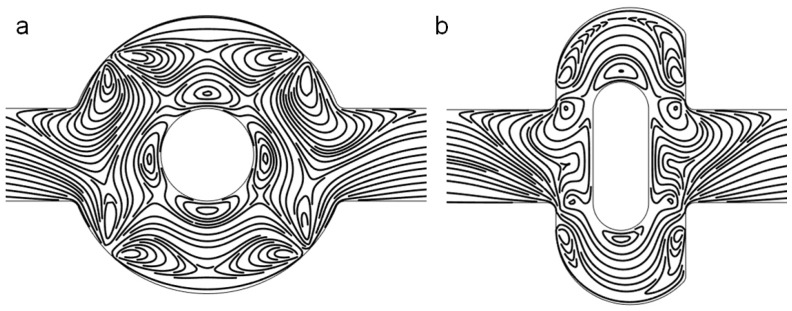
Fluid streamlines in (**a**) the original chip and (**b**) revised chip in the channel when the device uses the potentials $V_{0}=1$ V and the inlet velocity $U_{0}=10^{-3}$ m/s.

**Figure 4:**
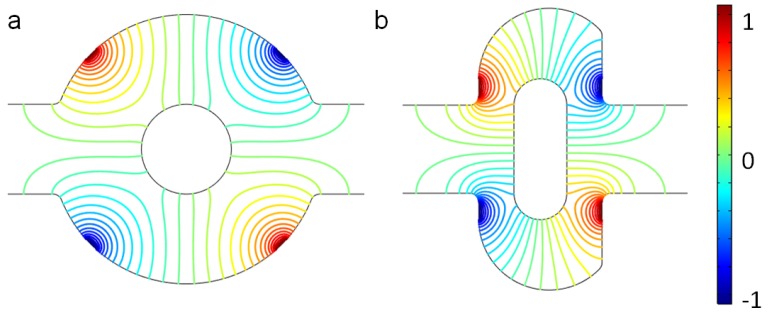
Electric potential lines in (**a**) the original chip and (**b**) the revised chip when the device uses the potentials $V_{0}=1$ V and the inlet mean velocity $U_{0}=10^{-3}$ m/s.

**Figure 5:**
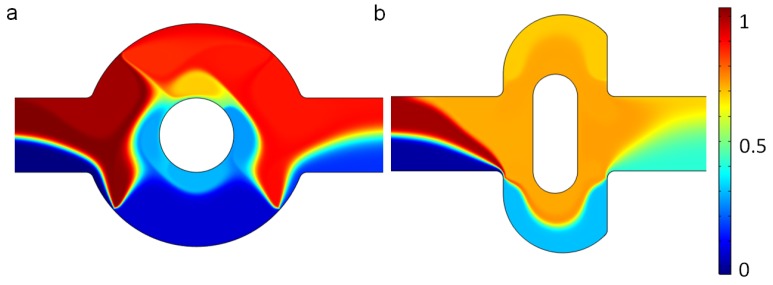
The concentration surface plot of (**a**) the original chip and (**b**) the revised chip when the device uses the potentials $V_{0}=1\;V$ and the mean inlet velocity $U_{0}=10^{-3}$ m/s. The unit for the concentration is mol/m${}^{3}$.

When the electric field is applied, the fluctuations in the flow increase considerably, as shown by the streamlines in Figure 3, due to the electroosmotic flow. In both mixers, there are some vortices near the electrodes. The applied voltage for both mixers is $V_{0}=1$ V, which generates a uniform electric intensity in the channel (Figure 4). Comparing the electric potential lines in the channels of the mixer, it is found that the nonuniformity is reinforced by the lateral structure of the revised chip due to the asymmetry, which can also be easily manufactured by standard photolithography [6]. Therefore, the rotating vortices caused by the electroosmotic flow due to the electrical field (which disturbs the main flow) is much more irregular near the electrodes in the revised chip, compared with the original chip (as shown in Figure 3). The vortices in the channel will fold and stretch the fluid element of two reagents, inducing an efficient mixing in the mixer (Figure 5). Furthermore, the range of influence for the vortex of the revised chip is stronger than that of the original mixer. From Figure 3, it can be seen that the vortex of the revised chip extends further into the main channel than that of the original chip. Figure 5 qualitatively illustrates the concentration before and after the expansion part of channel. Before the expansion part, the concentration of the two mixers is the same, while the two streams are much more homogeneous for the mixer with the lateral structure than the original mixer near the outlet, as observed in Figure 5.

### 3.2. Mixing Efficiency

**Figure 6:**
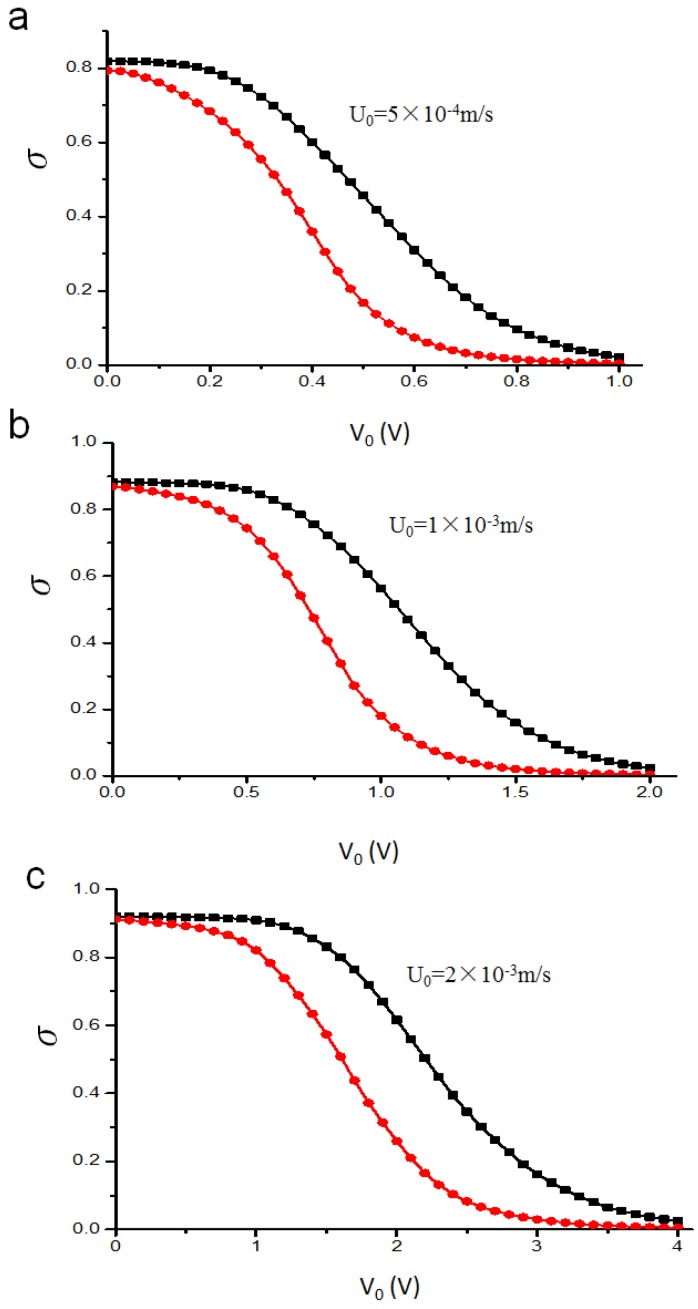
The mixing efficiency index *σ* versus the potential $V_{0}$, where the inlet mean velocity $U_{0}$ is (**a**) $5\times 10^{-4}$ m/s; (**b**) $1\times 10^{-3}$ m/s; (**c**) $2\times 10^{-3}$ m/s. Black line with square symbols: original chip; Red line with circular symbols: revised chip.

**Figure 7:**
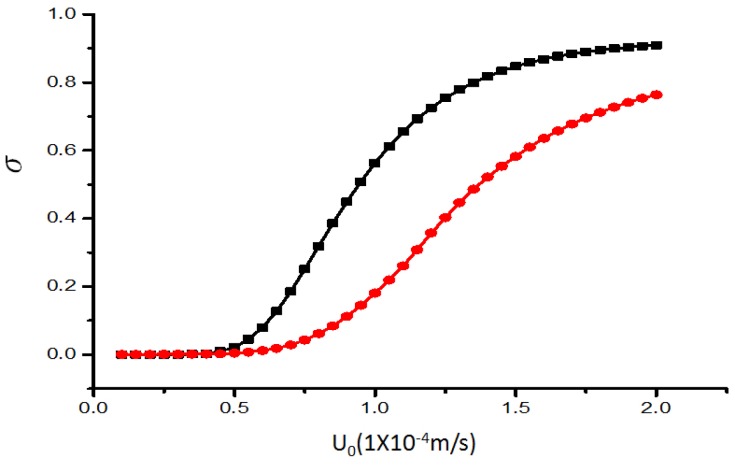
The mixing efficiency index *σ* of mixer versus the inlet mean velocity $U_{0}$ for $V_{0}=1$ V. The horizontal and vertical axes depict the inlet mean velocity $U_{0}$ and mixing efficiency index *σ*, respectively. The unit for the inlet mean velocity $U_{0}$ is $1\times 10^{-4}$ m/s. Black line with square symbols: original chip. Red line with circular symbols: revised chip.

In this part, we calculate the mixing efficiency index *σ* quantitatively based on the corresponding simulation result for the two mixers, considering the electrical potential and flow rate while the mixing efficiency index *σ* of 1 indicates unmixed fluids, and a mixing efficiency index *σ* of 0.0 indicates complete mixing. As the Smoluchowski slip velocity is proportional to the electric field intensity, the mixing level will be influenced as a result of enhanced disturbance induced in the microfluidic channel (Figure 6). Moreover, the mixing behavior will vary with the inlet mean velocity, because the mixing time will decrease with increasing flow rate (Figure 7).

Figure 6 compares the mixing efficiency index *σ* near the outlet along with the potentials $V_{0}$ for three different inlet mean velocities: (a) $5\times 10^{-4}$ m/s, (b) $1\times 10^{-3}$ m/s, (c) $2\times 10^{-3}$ m/s, in both the original and the revised mixer. From the result, it is found that the higher levels of mixing can be achieved when the electrical intensity increases for both mixers. Two species almost achieve complete mixing from poor mixing when the potentials $V_{0}$ raise from 0 to 1 V (2 V and 4 V), as shown in Figure 6a (Figure 6b and Figure 6c). However, the revised chip will improve the mixing of the samples much further as the electric potential $V_{0}$ is increased. This means that the stirring effect in the original mixer caused by EOF is less significant than the mixer with the lateral structure to substantially enhance mixing performance for the original mixer at the same $V_{0}$.

The mixing efficiency index *σ* near the outlet is shown as a function of the inlet mean velocity $U_{0}$ at the electrical potential $V_{0}=1$ V of the two mixers in Figure 6. For both mixers, the mixing effect will weaken when the flow rate is increased, due the reduction of mixing time, as manifested in Figure 7. This holds true for all values of $U_{0}$, though the proposed mixer outperforms the original one.

## 4. Conclusions

An efficient electroosmotic micromixer is designed by improving an existing electroosmotic micromixer with an asymmetric lateral structure. The mixing is achieved by the additional fluctuations in the flow due to this structure with the EOF generated by the electric field. The simulation results suggest that this enhanced mixer folds and stretches fluid samples in the channel more effectively than the original mixer. The influences of the electric potential applied and the flow rate are investigated to compare the performance of the two mixers. In all parametric studies, the modified structure considerably outperforms the original mixer. The results also indicate that the reagents can reach complete mixing in the enhanced mixer with lower potential and higher throughput compared to the original mixer. An experimental verification of the present numerical analysis is necessary for actual application of the proposed mixer, and will be the subject of future investigation.
